# Preparation and Characterization of Crosslinked Electrospun Gelatin Fabrics via Maillard Reactions

**DOI:** 10.3390/ma16114078

**Published:** 2023-05-30

**Authors:** Duangkamol Dechojarassri, Ryota Kaneshige, Hiroshi Tamura, Tetsuya Furuike

**Affiliations:** 1 Faculty of Chemistry, Materials and Bioengineering, Kansai University, Osaka 564-8680, Japan; 2 Organization for Research and Development of Innovative Science and Technology (ORDIST), Kansai University, 3-3-35 Yamate-cho, Suita, Osaka 564-8680, Japan

**Keywords:** crosslinker, GlcNAc, methylglyoxal

## Abstract

In this study, nonwoven gelatin (Gel) fabrics crosslinked using *N*-acetyl-D-glucosamine (GlcNAc) were characterized and compared with those crosslinked using methylglyoxal (MG) and by thermal dehydration. We prepared Gel with 25% concentration along with Gel/GlcNAc and Gel/MG with a GlcNAc-to-Gel ratio of 5% and MG-to-Gel ratio of 0.6%. A high voltage of 23 kV, solution temperature of 45 °C, and distance of 10 cm between the tip and the collector were applied during electrospinning. The electrospun Gel fabrics were crosslinked by heat treatment at 140 and 150 °C for 1 d. The electrospun Gel/GlcNAc fabrics were treated at 100 and 150 °C for 2 d, while the Gel/MG fabrics were heat-treated for 1 d. The Gel/MG fabrics exhibited higher tensile strength and lower elongation than the Gel/GlcNAc fabrics. Overall, Gel/MG crosslinked at 150 °C for 1 d showed a significant enhancement in tensile strength, high hydrolytic degradation, and excellent biocompatibility, with cell viability percentages of 105 and 130% at 1 and 3 d, respectively. Therefore, MG is a promising Gel crosslinker.

## 1. Introduction

Gelatin (Gel) is a protein derived from the partial hydrolysis of collagen triple helix molecules. It is mainly obtained from the connective tissue of the skin and bones of animals (cows, pigs, and fish). Because it is a bioabsorbable medical material with excellent properties and is a safe bio-adsorbent, Gel is applied mainly in the food industry as food packaging materials [1,2] and in biomedical materials such as scaffolds for articular cartilage; and in vascular, neural, and tendon tissue engineering [3,4,5,6,7,8,9]. Several studies report the development of Gel-based scaffolds and demonstrate the suitability of porous Gel scaffolds for fibroblast cultures [10,11]. Therefore, the production of Gel-based nanofibers via electrospinning has increased significantly over the last decade [12,13,14,15,16]. However, despite the high surface area to volume ratio of Gel nanofibrous mats, their poor mechanical strength and water resistance hinder their practical use. Consequently, several types of crosslinkers have been studied and reported to overcome these problems [17,18,19].

Glutaraldehyde (GTA) is mainly used as a Gel crosslinker in solution and under vapor conditions. However, the middle layers of the Gel membrane are poorly crosslinked during the large-scale production of Gel fabrics crosslinked with GTA vapor [17]. Thermal dehydration was also reported to occur during Gel crosslinking [18]. Moreover, various sugars such as glucose, sucrose, and fructose have been proposed as crosslinkers for Maillard reactions [19,20,21]. The Maillard reaction forms a Schiff base between an amino group and a reducing sugar in a protein. After Amadori rearrangement via numerous intermediates, the Schiff base reacts with other proteins to produce antibacterial effects. The reaction produces melanoidins with excellent oxidizing properties [21]. Etxabide et al. found that the Maillard reaction occurred in a Gel film through the addition of glucose, lactose, and ribose with thermal crosslinking at 105 °C [22]. Saiimon et al. prepared nanofibrous Gel scaffolds by electrospinning from aqueous acetic acid and crosslinking by adding glucose and heating at 170–175 °C for 3 h. They found that the elastic modulus of the Gel scaffolds increased from 0.3 to 1.1 GPa when 15% glucose was added [23]. In our previous work, GlcNAc was used as a reducing sugar for the Maillard reaction to crosslink nonwoven Gel nanofibers; it was heat-treated at 120 °C for 2 d (Gel/GlcNAc-120 °C fabrics) and was compared with the fibers obtained via treatment with GTA vapor (Gel/GTA fabrics). The results revealed that the tensile strength of the Gel/GlcNAc-120 °C fabrics was higher than that of the Gel fabrics but lower than that of the Gel/GTA fabrics [24].

Methylglyoxal (MG), an intermediate glycation product of the Maillard reaction, has been proposed for use as a Gel crosslinker via the Maillard reaction with amino acids in proteins to form crosslinks and improve the Gel’s mechanical properties. Although several polysaccharides have been investigated for Gel crosslinking by the Maillard reaction, few studies have been performed on the use of both GlcNAc and MG as crosslinkers in Gel nanofibers. Recently, MG was proven to be a crosslinker for the Gel, but in the hydrogel rather than the fiber form [25]. Therefore, this study focused on preparing nonwoven Gel fabrics crosslinked with GlcNAc, MG, and under thermal dehydration. The tensile properties, hydrolytic degradation, and biocompatibility of the fabrics were also investigated.

## 2. Materials and Methods

### 2.1. Materials

Cowhide-derived gelatin HGS-200 (Gel, Mw = 100,000, Lot No. 191112) was purchased from Koei Chemical Co., Ltd. (Hyogo, Japan). Phosphate-buffered saline (PBS) powder, *N*-acetyl-D-glucosamine (GlcNAc), and methylglyoxal (MG) solution (40% in H_2_O) were purchased from Wako Pure Chemical Industries Ltd. (Osaka, Japan). L929 mouse fibroblast Eagle’s medium (Lot No. 751810) was obtained from Nihon Pharmaceuticals Co., Ltd. (Tokyo, Japan). Fetal bovine serum was purchased from Biowest (Lakewood Ranch, FL, USA). TM Green CMFDA Cell Tracker (Lot No. 2089921) was purchased from Thermo Fisher Scientific Inc. (Osaka, Japan). Cell Counting Kit-8 (Dojindo Laboratories Co., Ltd., Kumamoto, Japan) and L929 mouse fibroblasts (RCB1451) were obtained from the Riken BRC Cell Bank (Tsukuba, Japan).

### 2.2. Preparation of Gel Solution

An amount of 10 g of Gel was added to 30 g of deionized (DI) water and stirred at 45 °C in a water bath until the solution was homogeneous. The mixture was incubated in a hot water bath for 30 min. Subsequently, it was defoamed by standing in an ice bath and remelting at 45 °C. The obtained solution containing 25% Gel was used as the dope solution.

### 2.3. Preparation of Gel/GlcNAc Solution and Gel/MG Solution

First, 0.5 g of GlcNAc was added to 29.5 g of DI water and then stirred at 45 °C in a water bath until a homogeneous solution was obtained. Next, 10 g of Gel was added to the obtained GlcNAc solution to prepare a 5% GlcNAc-to-Gel solution. The mixture was stirred under the same conditions until it became uniform. Subsequently, the mixture was incubated in a hot water bath for 30 min. Finally, the Gel/GlcNAc solution was defoamed by standing in an ice bath, remelted at 45 °C, and used as a dope solution.

**Figure 1:**
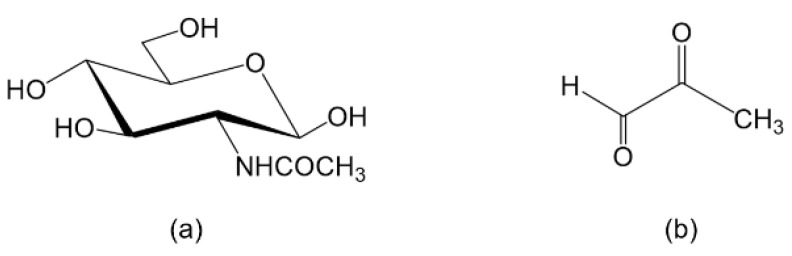
Chemical structures: (**a**) *N*-acetyl-D-glucosamine (GlcNAc) and (**b**) methylglyoxal (MG).

The preparation of the Gel/MG solution was similar to that of the Gel/GlcNAc solution. First, 0.17 g of the MG solution was added to 29.83 g of DI water and stirred at 45 °C in a water bath until the solution was homogeneous. Subsequently, 10 g of Gel was added to the prepared MG solution, with an MG-to-Gel ratio of 0.6%. The chemical structures of GlcNAc and MG are shown in Figure 1.

### 2.4. Preparation of Nonwoven Gel Nanofibers by Electrospinning Technique

**Figure 2:**
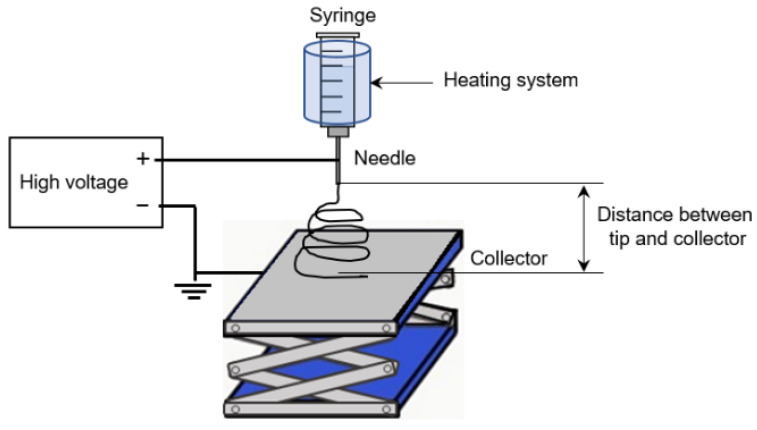
Electrospinning apparatus.

The nonwoven Gel nanofibers were prepared based on our previous study [24]. The electrospinning apparatus contained four essential components: a syringe as the dope solution container, conductive substrate as the ground, high-voltage supply, and heating system. Aluminum foil was spread over the conductive substrate and placed vertically downward from the nozzle of the electrospinning apparatus. The dope solution (5 mL) was then added to the syringe. The positive electrode was connected to the syringe, and the negative electrode to the aluminum foil. A high voltage of 23 kV, current density of 80 µA, solution temperature of 45 °C, and distance of 10 cm between the tip and the collector were applied. The electrospinning apparatus was set up as shown in Figure 2.

### 2.5. Crosslinking by Thermal Dehydration

The nonwoven Gel fabrics were allowed to stand at crosslinking temperature of 140 and 150 °C for 1 d.

### 2.6. Crosslinking by the Maillard Reaction

**Figure 3:**
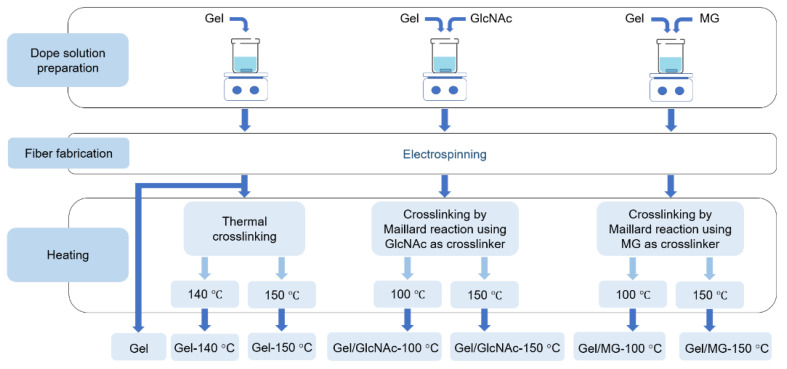
Sample preparation scheme.

The nonwoven Gel/GlcNAc fabrics were allowed to stand at crosslinking temperatures of 100 and 150 °C for 2 d. By contrast, the nonwoven Gel/MG fabrics were allowed to stand at crosslinking temperatures of 100 and 150 °C for 1 d. The sample names and preparation conditions are listed in Table 1. The sample preparation schemes for the different methods are shown in Figure 3.

### 2.7. Morphology of Nanofibers

FE-SEM measurements were performed using a scanning electron microscope (JSM6700; JEOL, Tokyo, Japan). The morphologies of the samples were observed before the PBS solution-resistance test. Each sample was vacuum-dried for 1 d and coated with platinum for 200 s before observation. The diameter distribution and average diameter of the fibers were determined from 100 fibers in the SEM images using ImageJ software.

### 2.8. Fourier Transform Infrared (FTIR) Spectroscopy Measurement

Fourier transform infrared spectroscopy (FTIR; FT/IR-4200, JASCO, Tokyo, Japan) was performed for the qualitative analysis of each sample using the KBr method. Before measurement, the finely chopped fibers were dried in a vacuum oven for 1 d. A measurement range of 4000–400 cm^−1^, accumulation count of 64, and resolution of 4.0 cm^−1^ were used.

### 2.9. Tensile Properties of Nanofibers

The tensile properties were measured using a universal testing machine (STA-1150; A&D Co., Ltd., Tokyo, Japan) at a tensile speed of 20 mm/min. First, the sample was cut to a width and length of 5.0 and 30.0 mm, respectively. Both ends of the 10.0 mm-length were then fixed to cardboard with an instant adhesive. Since the thickness of the sample was difficult to measure, the strength of the sample (in units of cN) was reported instead. The average strength and elongation values were obtained from the results for five pieces.

### 2.10. PBS Solution-Resistance Test

A PBS solution-resistance test was performed to examine hydrolytic degradation. A 0.067 M PBS solution was prepared by dissolving the PBS powder in DI water. The initial weight of the nanofibers was determined and noted as *W*_0_. Subsequently, the samples were immersed in the PBS solution and left at 37 °C for 1, 3, 5, 7, 14, 21, and 28 d. The samples were removed and dried at 100 °C for 1 d. The dry weights of the samples were measured. The remaining weight (%) at each point in time point was calculated using Equation (1):

Remaining weight (%) = (*W*_t_/*W*_0_) × 100 (1)

where *W*_t_ (g) and *W*_0_ (g) are the sample weights in the wet and dried states, respectively. The average value and standard deviation were calculated from five measurements and reported.

### 2.11. Cell Culture

Each nonwoven fabric was cut into a circle with a diameter of 7.0 mm and sterilized using UV irradiation for 1 h. The cells were subsequently immersed in serum-free EMEM overnight and allowed to stand in a 96-well plate. L929 mouse fibroblasts were prepared at a density of 1.0 × 10^4^ cells/well. Viable cells were fluorescently stained using Cell Tracker Green CMFDA and observed under a fluorescence microscope. The morphology was observed after 1 and 3 d. A Cell Counting Kit-8 was used to count the number of viable cells. The absorbance at 450 nm was measured using a TECAN Infinit F50 spectrophotometer (Männedorf, Switzerland). The cell viability was calculated using a calibration curve for various viable cell concentrations. The average value and standard deviation were calculated for three experiments and reported.

## 3. Results and Discussion

### 3.1. Maillard Reaction Mechanism of Gel by GlcNAc and MG

**Figure 4:**
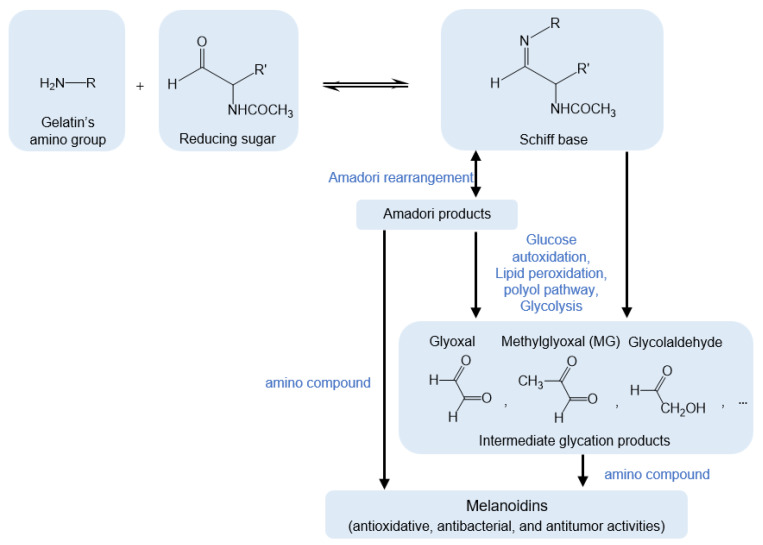
Maillard reaction scheme.

The Maillard reaction scheme for Gel using GlcNAc and MG is shown in Figure 4. First, the amino group of Gel interacts with a reducing sugar and the carbonyl group of GlcNAc to produce a Schiff base [24]. Subsequently, Amadori products are formed via Amadori rearrangement. Meanwhile, intermediate glycation products can be generated via another pathway involving glucose autoxidation, lipid peroxidation, the polyol pathway, and glycolysis [25,26]. After obtaining the Amadori products, the reaction continues via two pathways. In the first pathway, the Amadori products are converted directly to melanoidins by reacting with amino compounds. In the second pathway, Amadori products are converted to intermediate glycation products, including MG, and then to melanoidins (brown nitrogenous polymers and copolymers) by reacting with amino compounds [21]. Herein, the intermediate glycation product and a precursor of melanoidins were added to the Gel dope solution as a Gel crosslinker. The nonwoven Gel crosslinked by GlcNAc was compared with that obtained using MG and thermal dehydration.

**Figure 5:**
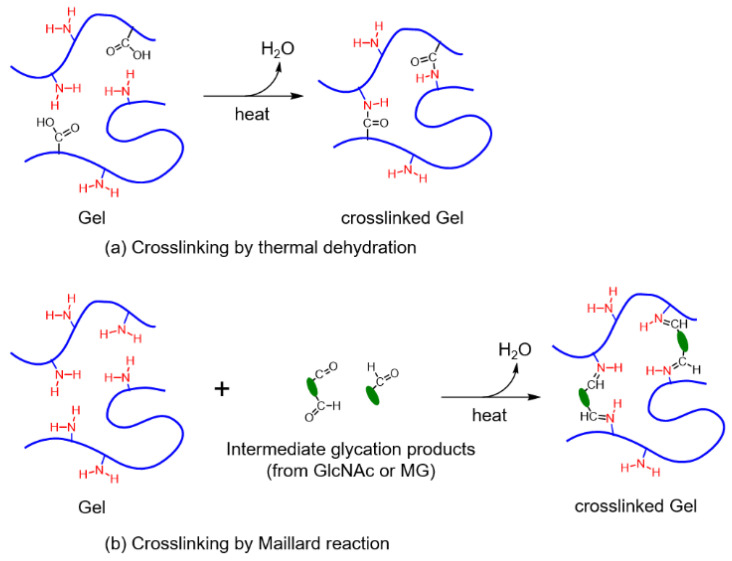
Examples of Gel-crosslinked structures.

Figure 5a shows that the crosslinked Gel is obtained under high temperatures, leading to the reaction between the amino and carboxyl groups in Gel. By contrast, the crosslinked structure obtained by the Maillard reaction is due to the reaction between the amino group of gelatins and the carbonyl group of GlcNAc or MG, as shown in Figure 5b.

### 3.2. Morphological Observation

**Figure 6:**
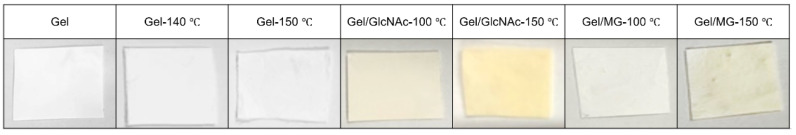
Digital photographs of Gel fabrics.

The digital photographs of the Gel fabrics under different conditions are shown in Figure 6. The Gel, Gel-140 °C, and Gel-150 °C fabrics are white. In addition, shrinkage of the Gel fabrics is observed after heat treatment. By contrast, the Gel/GlcNAc-100 °C, Gel/GlcNAc-150 °C, Gel/MA-100 °C, and Gel/MA-150 °C fabrics are light yellow, yellow, off-white, and a light yellow-flame, respectively. The color changes in these fabrics indicate melanoidin formation via the Maillard reaction [24]. Kwak et al. found that Gel nanofibers crosslinked with either glucose or fructose turned yellow after heat treatment at 100 °C for 4 h [21]. Among all samples, swelling of the Gel/GlcNAc-150 °C fabrics was visibly observed, possibly because of the hydrophilic properties of GlcNAc [24].

**Figure 7:**
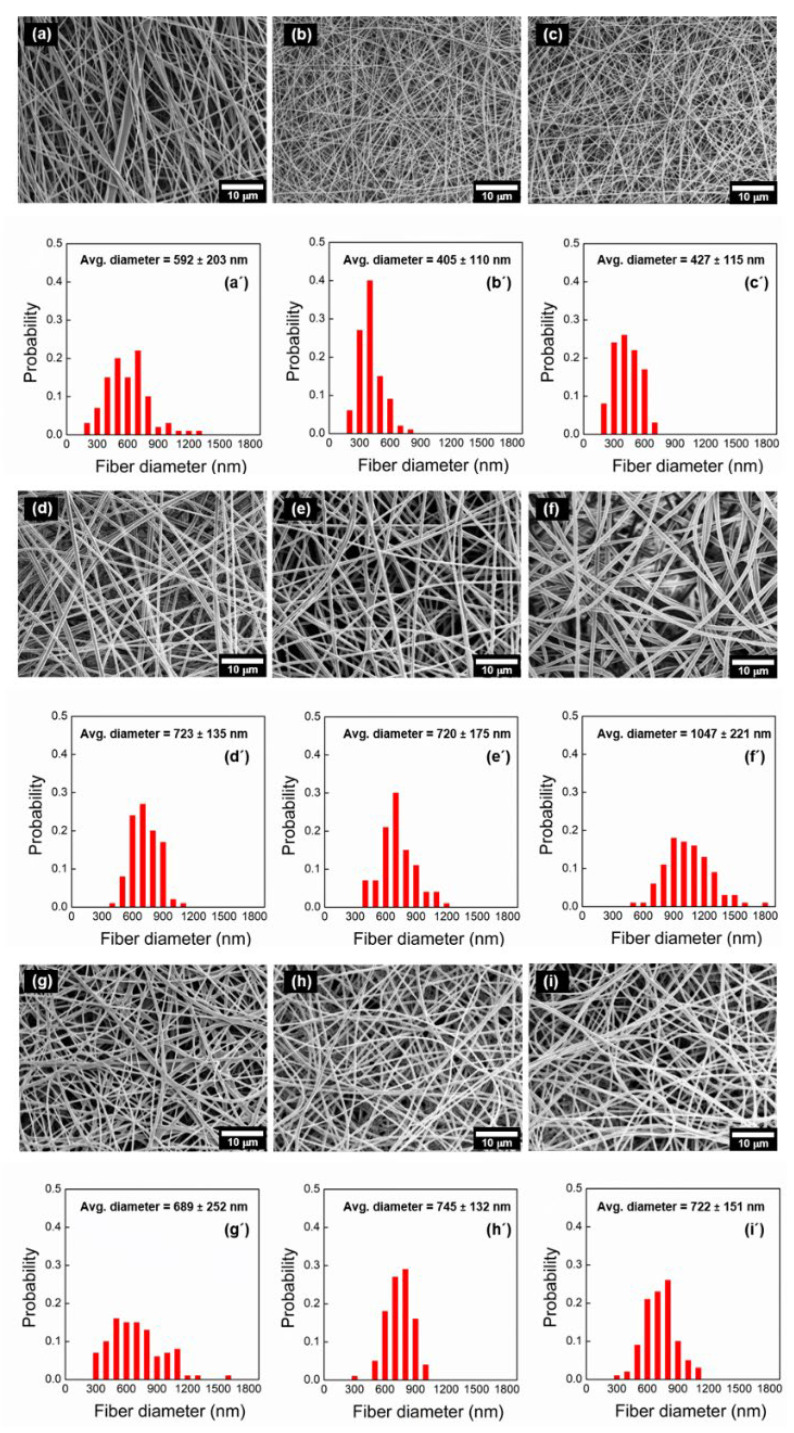
(**a**–**i**) SEM images of Gel fabrics and (**a′**–**i′**) size distribution of the fiber diameters. (**a**,**a′**) Gel fabrics, (**b**,**b′**) Gel-140 °C fabrics, (**c**,**c′**) Gel-150 °C fabrics, (**d**,**d′**) Gel/GlcNAc fabrics, (**e**,**e′**) Gel/GlcNAc-100 °C fabrics, (**f**,**f′**) Gel/GlcNAc-150 °C fabrics, (**g**,**g′**) Gel/MG fabrics, (**h**,**h′**) Gel/MG-100 °C fabrics, (**i**,**i′**) Gel/MG-150 °C fabrics.

Figure 7 shows the SEM images of the Gel, Gel/GlcNAc, and Gel/MG fabrics before and after heat treatment and their fiber diameter distributions. The Gel, Gel/GlcNAc, and Gel/MG fabrics are observed to be smooth and continuous with an applied voltage of 23 kV. The average fiber diameters of the Gel/GlcNAc and Gel/MG fabrics are larger than those of Gel because the viscosity of Gel increased after the addition of GlcNAc or MG. These findings are consistent with those of previous studies [27,28]. After heat treatment, the average fiber diameters of the Gel-140 °C and Gel-150 °C fabrics are smaller than those of the fabrics without heat treatment (Gel). The decrease in the fiber diameters of the Gel-140 °C and Gel-150 °C fabrics is attributed to thermal hydration during heat treatment. Meanwhile, the Gel/GlcNAc-100 °C fabric exhibits an average fiber diameter of 720 nm, similar to that of Gel without heat treatment. After the heat treatment of Gel/GlcNAc at 150 °C for 1 d, the average fiber diameter increases dramatically to 1047 nm. Unlike the Gel crosslinked with GlcNAc, the average fiber diameters of the Gel/MG-100 °C and Gel/MG-150 °C fabrics are slightly larger than those of the Gel/MG fabrics.

### 3.3. Characterization by FTIR Analysis

**Figure 8:**
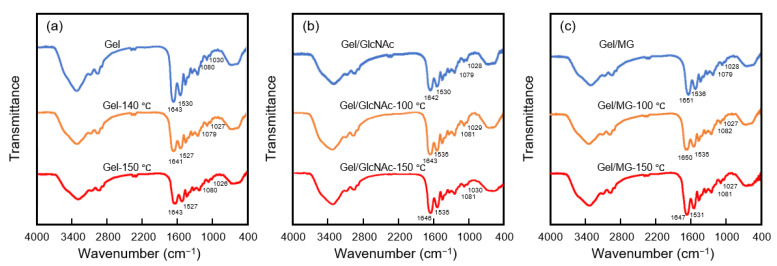
FTIR spectra of various fabrics: (**a**) Gel, Gel-140 °C, and Gel-150 °C fabrics; (**b**) Gel/GlcNAc, Gel/GlcNAc-100 °C, and Gel/GlcNAc-150 °C fabrics; and (**c**) Gel/MG, Gel/MG-100 °C, and Gel/MG-150 °C fabrics.

The FTIR spectra of the Gel, Gel-140 °C, Gel-150 °C, Gel/GlcNAc, Gel/GlcNAc-100 °C, Gel/GlcNAc-150 °C, Gel/MG, Gel/MG-100 °C, and Gel/MG-150 °C fabrics are shown in Figure 8. In this work, pure Gel exhibited peaks at 1643 and 1530 cm^−1^, corresponding to the amide I (C=O stretching/hydrogen bonding coupled with COO^−^) and amide II bands (arising from the bending vibration of the N–H group and the stretching vibration of C–N group), respectively [1], which were similar to those observed for fish gelatin nanofiber [20]. The Gel nanofibers with and without sugar molecules exhibited overlapping peaks in the region of 1130–1000 cm^−1^, representing the stretching vibration of C–C and C–O and the bending vibration of the C–H group [20]. Siimon et al. found that the relative absorbance change of the peaks at 1081 and 1035 cm^−1^ associated with the vibrations of C–O mainly in glucose decreased after crosslinking by the Maillard reaction between the carbonyl group of glucose and the amino group of gelatins [29]. Lin et al. also found that the carbonyl group of glucose reacted with the amino group of Gel in a Gel-glucose glycated system, leading to the formation of the C–O–C ether linkage. This linkage was due to OH condensation and manifested as a peak in the 1050–1110 cm^−1^ range [30]. Kchaou et al. confirmed that the peak around 1150–1110 cm^−1^ of Gel film without heating shifted to 1093 cm^−1^ after adding glucose and heating at 90 °C for 24 h [2]. In addition, a strong peak corresponding to the stretching vibrations of the C–N group around 1630–1660 cm^−1^ was observed in Gel crosslinked by the Maillard reaction [31,32]. As shown in Figure 8, the relative absorbance changes of the peaks around 1080 and 1030 cm^−1^ after crosslinking are too weak to be clearly displayed in this work. However, the shift of IR bands around 1630–1660, 1080, and 1030 cm^−1^ is observed after crosslinking. Although these 2–4 cm^−1^ shifts are too small to conclude the occurrence of crosslinking by the Maillard reaction, the changes in the color of the samples crosslinked with GlcNAc and MG shown in Figure 6, imply that the Maillard reaction has occurred after heating through the addition of GlcNAc or MG to the Gel dope solution.

### 3.4. Mechanical Properties

**Figure 9:**
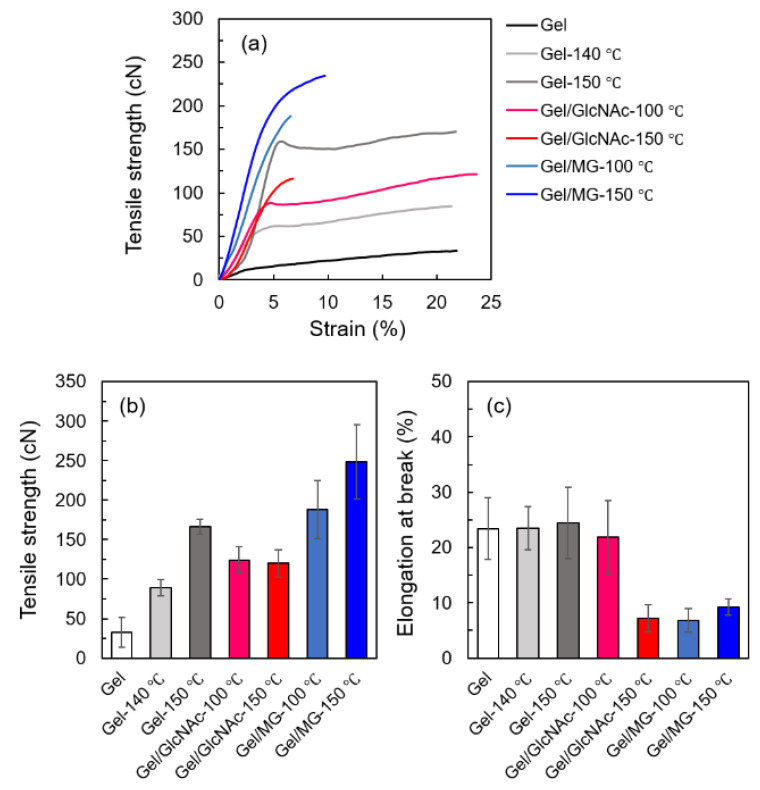
Tensile properties of each sample: (**a**) strength-strain curve, (**b**) tensile strength, and (**c**) elongation at break of Gel, Gel-140 °C, Gel-150 °C, Gel/GlcNAc-100 °C, Gel/GlcNAc-150 °C, Gel/MG-100 °C, and Gel/MG-150 °C fabrics.

The mechanical properties of Gel nanofibers can be enhanced by glucose crosslinking [23,33]. The strength-strain curve, tensile strength, and elongation at break of the Gel, Gel/GlcNAc, and Gel/MG fabrics before and after heat treatment are shown in Figure 9. The results indicated that the tensile strength dramatically increased after heat treatment, especially in the samples crosslinked with MG. By contrast, the elongation at break of the samples crosslinked with GlcNAc and MG tended to decrease. These findings were probably because a strong crosslink was established by the thermal crosslinking of GlcNAc and MG via the Maillard reaction, leading to a decrease in the elongation at break [21,24]. Interestingly, the tensile strength of Gel/MG-150 °C fabrics was 248.65 cN higher than that of the Gel fabrics crosslinked with GTA vapor reported previously [24].

### 3.5. PBS Solution Resistance

**Figure 10:**
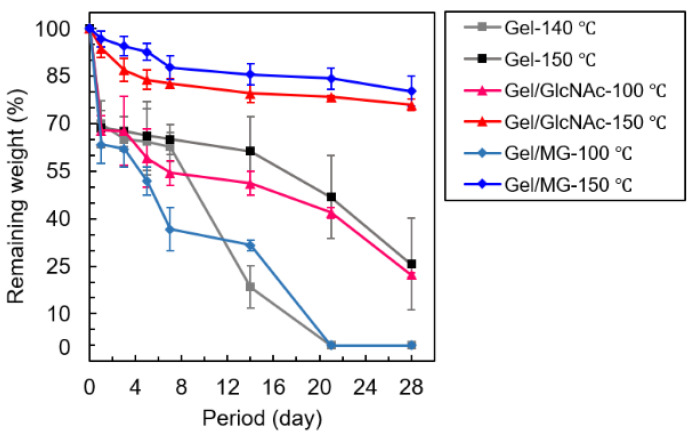
Weight ratio (%) of various fabrics vs. soaking period.

A PBS solution-resistance test was performed to examine hydrolytic degradation, which is an indicator of the durability of each sample under a wet stage. Figure 10 shows the remaining weight (%) plotted versus the soaking time. Since the nanostructure of the Gel fiber without any crosslinking rapidly dissolved in PBS, the result for the PBS solution resistance of the Gel nanofiber is not reported here. The Gel-140 °C and Gel/MG-100 °C fabrics dissolved completely in the PBS solution after 20 d, whereas the Gel-150 °C and Gel/GlcNAc-100 °C fabrics remained at approximately 30% weight at 28 d. Meanwhile, the Gel/MG-150 °C and Gel/GlcNAc-150 °C fabrics remained at approximately 80% weight at 28 d. These PBS solution resistance results can be explained by the degree of crosslinking, where a higher remaining weight corresponds to greater degree of crosslinking [21]. Kwak et al. found that the Maillard reaction of added sucrose led to the highest stability of the Gel nanofibers against hydrolytic degradation, followed by those of glucose and fructose [21]. Tang et al. found that nanofibers with a high degree of crosslinking maintained their structure in a wet environment [34]. From these results, it was concluded that the Gel/MG-150 °C fabrics showed the highest durability, followed by the Gel/GlcNAc-150 °C fabrics, Gel-150 °C fabrics, Gel/GlcNAc-100 °C fabrics, Gel/MG-100 °C fabrics, and Gel-140 °C fabrics.

### 3.6. Cell Culture

**Figure 11:**
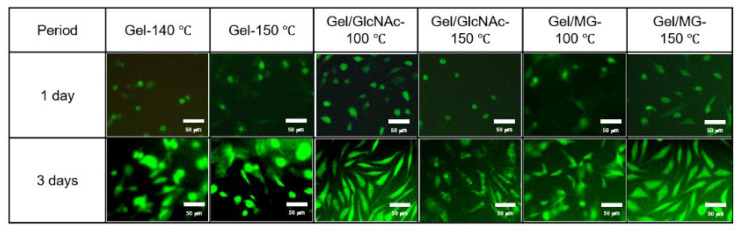
Representative fluorescence micrographs of live-stained Gel-140 °C, Gel-150 °C, Gel/GlcNAc-100 °C, Gel/GlcNAc-150 °C, Gel/MG-100 °C, and Gel/MG-150 °C fabrics at 1 and 3 d.

**Figure 12:**
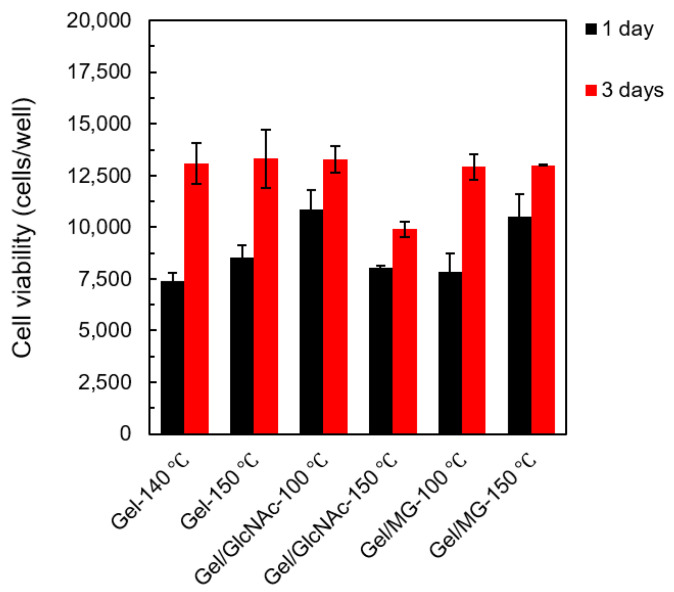
Cell viability of Gel-140 °C, Gel-150 °C, Gel/GlcNAc-100 °C, Gel/GlcNAc-150 °C, Gel/MG-100 °C, and Gel/MG-150 °C fabrics at 1 and 3 d.

For the cell culture, 1.0 × 10^4^ cells/well of L929 mouse fibroblasts were prepared as a cell model. The cell viability was calculated using a calibration curve for various viable cell concentrations. The average value and standard deviation were calculated from three experiments and reported. Representative fluorescence micrographs of live-stained fabrics and the cell viability of each fabric at 1 and 3 d are shown in Figure 11 and Figure 12, respectively.

At 3 d, it was observed that the cell number of every fabric was higher than at 1 d. Compared to the cell viability in the initial stage (1.0 × 10^4^ cells/well), it seems like Gel-140 °C, Gel-150 °C, Gel/GlcNAc-150 °C, and Gel/MG-100 °C fabrics killed the L929 mouse fibroblasts at the initial stage (1 d); by contrast, this was not observed with the Gel/GlcNAc-100 °C and Gel/MG-150 °C fabrics. Although the cell numbers decreased, the cell viability was equal to or higher than 85%, implying good biocompatibility for all fabrics. Among all samples, the Gel/GlcNAc-100 °C fabric exhibited enhanced cell growth, with a 109% increase in the cell viability after the initial stage and a 133% increase after 3 d. The Gel/MG-150 °C fabric exhibited the cell viabilities of 105 and 130% at 1 and 3 d, respectively. Compared to our previous work, the Gel/MG-150 °C fabric has better biocompatibility than those crosslinked with GTA vapor and GlcNAc-120 °C [24]. Based on these findings and the results for the mechanical properties and hydrolytic degradation, it can be concluded that the Gel/MG-150 °C fabric is the most suitable material for use as a scaffold.

Interestingly, an elongated cell morphology was clearly observed for all fabrics, especially for the fabric crosslinked by the Maillard reaction corresponding to previous work [5,6]. Yang et al. fabricated the aligned poly(L-lactic acid) (PLLA) nano-/micro-fibrous scaffolds using an electrospinning technique and Neonatal mouse cerebellum C17.2 stem cells (NSCs) with a density of 2.8 × 10^4^ cells/cm^2^ as a model cell. The direction of NSC elongation and neurite outgrowth was found to be parallel to the direction of fiber alignment [5]. Furthermore, Darshan et al. used the electrospinning technique to prepare a gelatin/poly(ε-caprolactone)/heparin (GPH) nanofiber membrane. They then immersed the membrane in basic human recombinant fibroblast growth factor (bFGF) to produce a GPH-bFGF scaffold. In the cell viability test, a 1 cm scaffold was rinsed with a cell culture medium in a 12-well plate, seeded with 3.0 × 10^5^ tenocytes in 20 μL cell suspension, and incubated at 37 °C. On day 7, more live cells with an elongated cell morphology using the GPH-bFGF scaffold were observed [6]. These findings were probably due to the very small pore size and pore shape produced by the electrospinning technique, which allowed the cell to grow along the pore direction or the fiber alignment.

## 4. Conclusions

This study successfully prepared crosslinked electrospun Gel fabrics with fiber diameters ranging from 400 to 1000 nm via Maillard reactions using either GlcNAc or MG and by heat treatment. The Gel fabric crosslinked with MG at 150 °C for 1 d exhibited improved tensile properties, with the strength increasing from 32.85 to 248.65 cN, and demonstrated a 130% increase in cell growth percentage after 3 d. These results suggest that this fabric has good tensile properties and excellent biocompatibility, making it a promising material for biomedical applications, mainly as scaffolds.

## Figures and Tables

**Table 1:** Sample names and their preparation conditions.

Sample Name	Dope Solution	Heat Treatment Condition
Gel	Gel	–
Gel-140 °C	Gel	140 °C for 1 d
Gel-150 °C	Gel	150 °C for 1 d
Gel/GlcNAc	Gel/GlcNAc	–
Gel/GlcNAc-100 °C	Gel/GlcNAc	100 °C for 2 d
Gel/GlcNAc-150 °C	Gel/GlcNAc	150 °C for 2 d
Gel/MG	Gel/MG	–
Gel/MG-100 °C	Gel/MG	100 °C for 1 d
Gel/MG-150 °C	Gel/MG	150 °C for 1 d

## Data Availability

Data will be made available on request.
